# Impact of interventions by a community pharmacist on care burden for people with dementia: development and randomized feasibility trial of an intervention protocol

**DOI:** 10.1186/s40814-022-01071-7

**Published:** 2022-06-02

**Authors:** Yoko Nanaumi, Atsushi Yoshitani, Mitsuko Onda

**Affiliations:** 1 Advance Pharma Research Office, 3-6-2 Ukyo, Nara, 631-0805 Japan; 2 Nara City Pharmaceutical Association, 768, Kidera-cho, Nara, 630-8306 Japan; 3 Faculty of Pharmacy, Osaka Medical and Pharmaceutical University, 4-20-1, Nasahara, Takatsuki City, Osaka Japan

**Keywords:** Dementia, Caregiver burden, Community pharmacist

## Abstract

**Background:**

Traditionally, the role of pharmacists has been to manage and monitor pharmacotherapy for patients with dementia. However, additional intervention by community pharmacists to collect and share patient information with other professionals may help reduce the care burden among caregivers. The aims of this study were to examine (1) the feasibility of a designed community pharmacist working procedure in dementia care and (2) the expected impact of pharmacist intervention on care burden.

**Methods:**

This was a randomized, open-label, parallel-group feasibility study, involving eight Nara City pharmaceutical association member pharmacies that provided consent to participate. These pharmacies were assigned to an intervention group or a control group at a 1:1 ratio. The subjects were patients with dementia and their primary caregivers that visited the participating pharmacies and provided consent to participate. Pharmacists in the intervention group actively collected information from the patients’ family physicians and care managers and intervened to address medication-related problems, while those in the control group only performed their normal duties. The primary endpoint was a change in the caregiver’s score on the Japanese version of Zarit Caregiver Burden interview (J-ZBI) from the baseline to after 5 months of follow-up. The changes in mean J-ZBI scores from the beginning to the end of the study period of the two groups were compared.

**Results:**

Obtaining consent from caregivers was certainly difficult, but possible. Pharmacists managed to fill out the survey form while practising pharmaceutical care. Totally, nine patients and nine caregivers in the intervention group and nine patients and eight caregivers in the control group completed the study. The changes in J-ZBI scores could be calculated for seven cases in the intervention group and five cases in the control group. The J-ZBI scores were found to decrease by 1.0 in the intervention group and increase by 3.0 in the control group.

**Conclusions:**

The protocol presented was considered feasible, but, the intervention process needs to be simplified in order to conduct a large study. Also, improvements are needed in the various survey forms and in the explanatory documents for caregivers. Although the sample size was small, the effect sizes suggested that community pharmacist interventions for patient with dementia may reduce the care burden for caregivers.

**Trial registration:**

UMIN000039949 (registration date: 1 April 2020, retrospectively registered)

**Supplementary Information:**

The online version contains supplementary material available at 10.1186/s40814-022-01071-7.

## Key messages regarding feasibility

- *What uncertainties existed regarding the feasibility?*
  The following uncertainties are assumed regarding feasibility:
  - Is it possible for pharmacists to obtain consent from caregivers to participate in this study?
  - Is it possible for pharmacists to provide counselling and record data based on various forms during their daily work?
  - Are the 22 J-ZBI items adopted as a measure of care burden appropriate for this study?
  - Is a 5-month follow-up period appropriate for assessing intervention outcomes?
- *What are the key feasibility findings?*
  - Obtaining consent from caregivers is certainly difficult, but possible.
  - Pharmacists can complete the survey form while practising pharmaceutical care.
  - Many J-ZBI items are time-consuming for caregivers.
  - The 5-month follow-up period was too short to obtain clear outcomes or to confirm that the problem had been resolved.
- *What are the implications of the feasibility findings for the design of the main study?*
  - The number of participants obtained per pharmacy was low, so the number of pharmacies needs to be increased.
  - Pharmacists’ opinions obtained by fax after the study was completed indicated that the protocol presented was considered feasible, but that improvements were needed in the various survey forms and in the explanatory documents for caregivers.
  - It was also noted that the intervention process needs to be simplified in order to conduct a large study.
  - Since the 22-item version of the J-ZBI is burdensome to fill out, it is recommended that an 8-item version be used the next time it is implemented.
  - The follow-up period should be extended to a minimum of 6 months.

## Introduction

It is estimated that more than 50 million people are currently living with dementia worldwide, a number predicted to increase to 152 million by 2050 [1]. In Japan alone, the number of dementia patients (prevalence rate) among the elderly over the age of 65 years is expected to reach 7 million (20%) by 2025 [2], and the burden of care is becoming a social problem [3–6]. The lack of social services [7], low level of education of caregivers (intelligibility) [6], low self-efficacy of patients [8], behavioural and psychological symptoms of dementia (BPSD) [9], and psychosocial factors, including family functioning and relationships, contribute to the increased burden of care [10]. In general, caregivers of patients with dementia are prone to compromised health and low quality of life due to the physical, psychological, and social burdens associated with caregiving, resulting from the long process and unpredictable outcomes [11–13]. The increased burden of caregiving also has a profound impact on the well-being of society as a whole. Meanwhile, several interventional studies of field-level measures aimed at reducing the burden of caregiving have reported effectiveness [11], including improving relations between family and caregiving staff [12] and enhancing caregiver self-efficacy [13].

Several countries have national-level strategies, such as the Prime Minister's Challenge on Dementia in England [14], for supporting people with dementia through multi-profession collaborations [15]. Few, like the German [15] and Canadian national dementia strategies [16], specify the response to the burden of caregiving. However, the function and role of the pharmacy and pharmacist in reducing the care burden have not been clearly positioned in the inter-profession collaboration framework of either of these strategies.

The Japanese National Strategy is a comprehensive strategy formulated by the Ministry of Health, Labour and Welfare in July 2017 to accelerate dementia measures (New Orange Plan) in the country [17]. This plan has unique characteristics compared to the national strategies of other countries. For instance, the need for ‘enhanced responsiveness to dementia’ by pharmacies and pharmacists is specified in the context of a regional comprehensive care system. Specifically, since pharmacists represent the health professionals most accessible to the community [18], the roles assigned to them include support for caregivers to reduce the burden of care, early detection of dementia, and management of pharmacotherapy [17].

Traditionally, the primary roles of pharmacies and pharmacists in the care of people with dementia have been to improve the management of pharmacotherapy [19–22] and adherence to medication regimens [23, 24]. It has been suggested that community pharmacists can optimize pharmacotherapy for BPSD by sharing patient information with their general practitioners [25]. A prospective cohort study previously conducted by the authors suggests that enhancing the collection of patient information by community pharmacists may help to reduce the burden of caregiving [26]. In addition, assessing the effectiveness of community pharmacists by monitoring caregiver burden has been initiated [27]. However, whether community pharmacist interventions contribute to a reduction in the burden of caregiving is not fully understood [28]. Therefore, the objectives of the current study were to examine (1) the feasibility of a developed community pharmacist working procedure in dementia care and (2) the expected impact of pharmacist intervention on care burden.

## Methods

### Trial design

The study was designed as a randomized, open-label, parallel-group, controlled feasibility study and was performed in accordance with the CONSORT statement (pilot of feasibility randomized trial) [29]. Since there is no effect size that can be used as a reference in estimating the sample size, this pilot study aims to find a reference value. When cluster randomized controlled trials are conducted in the future, the sample size should be calculated by correcting for the variation among pharmacies with an internal correlation coefficient.

### Participants

#### Pharmacies

Member pharmacies of the Nara City pharmaceutical association who attended the briefing session for this study and agreed to participate were included as working pharmacies.

#### Patients

Outpatients with dementia who were using one of the research cooperation pharmacies and receiving anti-dementia medications were included in the study. Written informed consent was obtained from the patients or their legal representatives. Individuals who did not provide consent to participate and those receiving home care services by pharmacists were excluded from the study. Home service recipients were excluded as their information may have been shared previously with the family physician and other related professionals, and interventions may have been advised accordingly.

### Caregivers

The primary caregiver of a person with dementia provided consent to participate in the study. As a part of their job, community pharmacists are required to identify the primary caregiver while providing the medication; this information is recorded in the patient’s profile. Accordingly, we obtained written consent from the primary caregiver to participate in the study when they visited the pharmacy.

### Randomization

Pharmacies were randomly assigned to a group by the study director according to a random computer-generated number table. Pharmacies were randomly assigned to the intervention or control group, in a 1:1 ratio, without employing block randomization. A biostatistician at the Osaka University of Pharmaceutical Sciences employed computer-generated random numbers for obtaining the random allocation sequence. Pharmacists in each collaborative pharmacy recruited and assigned participants.

### Recruitment

The participating pharmacists verbally introduced the programme to those visiting the pharmacies. They approached those who were interested in the trial, explained its contents, and obtained written informed consent for their participation in the current trial. Participants were recruited from November 1–30, 2018; the follow-up period for each patient in the trial was at 5 months.

### Blinding

The intervention was assumed to be behavioural; thus, the pharmacists were not blinded. However, the participants and analysts were blinded to group allocation.

### Intervention group

Intervention group collected patient information during a patient/caregiver visit, using the ‘Basic Information Sheet’ shown in Additional file 1: Appendix 1. The ‘Basic Information Sheet’ includes information collected during the first visit (patient’s gender, age, primary caregiver, residential environment, prescriptions from medical institutions for the treatment of dementia, and prescriptions from other medical institutions), as well as information collected during each subsequent visit (details of any prescription changes made since the previous visit, and details of any actions taken by the family physician or care manager; Additional file 1: Appendix 1). The main caregivers were also asked to complete the Japanese version of the Zarit Caregiver Burden interview (J-ZBI) published by Sankyobo (use registration number: 1494J-ZBI). The J-ZBI is a self-administered questionnaire aimed at measuring a caregiver’s perception of the burden of caregiving. The questionnaire is composed of 22 items, scored on a 5-point scale ranging from 0 to 4; the smaller the total score, the lower the burden of caregiving experienced by the individual [30]. In addition, the main caregiver was counselled by the pharmacist based on the ‘Understanding of dementia survey sheet’ shown in Additional file 1: Appendix 2. Scores were recorded as binary values (1: yes, 0: no). The survey sheet was a comprehension test used at Kishiwada-city in 2017 as the Dementia Supporter Training Program prepared by the Ministry of Health, Labour and Welfare. It consisted of five statements to be marked as yes or no: (1) ‘The progression of dementia can be slowed by treatment and care’, (2) ‘The progression of symptoms of dementia can be slowed down by medication’, (3) ‘Dementia has “peripheral symptoms” in which behavioural and mental disorders occur’, (4) ‘There are several types of dementia, including Alzheimer's type and multi-infarct type’, and (5) ‘Measures against lifestyle related diseases are effective in preventing dementia’. The higher the total score, the better the caregiver’s understanding of the condition (Additional file 1: Appendix 2).

Next, pharmacists used the ‘Grasp problems sheet’ shown in Additional file 1: Appendix 3 to counsel the primary caregivers and assess any medication-related problems. Based on previous studies [31, 32], this sheet consisted of questions addressing the caregiver’s awareness about the medication (6 items), collaboration with healthcare providers regarding medication (3 items), motivation to access and utilize information regarding medication (5 items), and agreement with patient taking medications and their fit with the patient’s lifestyle (3 items). The aim was to identify and assess issues by setting assessment criteria for each question (Additional file 1: Appendix 3).

The pharmacists sent forms for the request of information to the family physician (Additional file 1: Appendix 4) and care manager (Additional file 1: Appendix 5) to actively collect patient information. ‘The form for request of information’ delivered to the family physician asked for the name of the primary disease, information on clinical test data used for diagnosis, the level of cooperation with other occupations, guidance and treatment plan given to the family members and caregivers, and any requests for the pharmacy. A care manager is a qualified healthcare provider who develops a long-term care service plan and communicates and coordinates with care service providers upon request from the caregivers and families, as part of the Japanese long-term care insurance system implemented in 2000. The request form to care managers asked for the main disease leading to the need for long-term care insurance service, degree of care required, results of care assessment and policy of care plan, level of cooperation with the family physician, problems with care and future policy, and any requests for the pharmacy (Additional file 1: Appendix 4 and 5).

The pharmacist shared information with the family physician, proposed prescription changes, changed dispensing methods, provided patient compliance instructions, requested consultations, and provided information on the issues identified in the ‘Grasp problems sheet’, based on information obtained in ‘The forms for request of information’. The follow-up period was 5 months, and during the final visit, the patients/caregivers were counselled again based on the ‘Basic Information Sheet’ and ‘Grasp problem sheet’. The caregivers were asked to complete the J-ZBI and the ‘Understanding of dementia survey sheet’ once again.

### Control group

Pharmacists in pharmacies assigned to the control group also collected information using the ‘Basic Information Sheet’, ‘Understanding of dementia survey sheet’, J-ZBI, and ‘Grasp problems sheet.’ However, they did not participate in aggressive information gathering using the ‘Forms for request of information.’

### Feasibility outcomes

With regard to feasibility outcomes, the following items were set out.

1. Possibility of obtaining consent from caregivers
2. The feasibility of conducting problem identification, counselling, and data collection
3. The appropriateness of adopting J-ZBI 22items for this study
4. Appropriateness of the follow-up period

### Outcomes

(1) The primary outcome was the change in the burden of care, and the primary endpoint was the change in the primary caregiver’s J-ZBI score. (2) Secondary outcomes included changes in caregiver comprehension of dementia, prescription changes, and action taken by the attending physician or care manager. The endpoint for changes in the caregiver’s understanding of the condition was the change in the primary caregiver’s score in the ‘Understanding of dementia survey sheet’ (Additional file 1: Appendix 2). The endpoint for prescription changes was the number of patients for whom the duration of a particular treatment was changed, or a medication was changed, added, or stopped. The endpoint for assessing the presence or absence of the action taken by the family physician or care manager was the number of patients who had some of their family physician or care manager approach pharmacists (this was referred to as ‘action’).

### Sample size

Calculating the sample size was difficult because there were no previous studies and no effect size could be established. Therefore, based on the information provided by the pharmacist, we aimed to recruit two patients and two caregivers per pharmacy, securing 16 patients per arm.

### Statistical analysis

The mean differences in J-ZBI scores and dementia comprehension scores from the baseline to after 5 months of follow-up were compared between the two groups followed by calculation of 95CI and effect size [33]. Due to the small sample size, hypothesis testing should be interpreted with caution and treated as preliminary. In addition, the number of prescription changes and actions taken by family physician and/or care manager was compared between the groups. SPSS statistics software (IBM, Armonk, NY, USA) was used for analysis.

### Assessment of feasibility

After the study was completed, the participating pharmacists were asked by fax for their opinions on the feasibility of the study, issues to be addressed, and future improvements.

### Ethical considerations

**Fig. 1:**
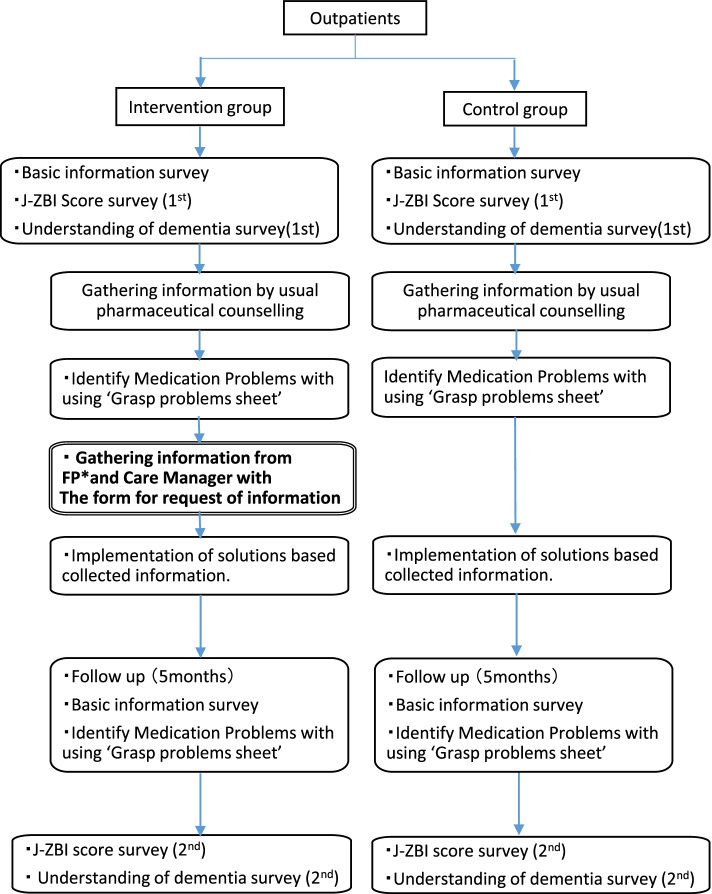
Study protocol for the two groups

The study design was approved by the Research Ethics Review Committee of Osaka Medical and Pharmaceutical University (approval number: 0048). Protocol registration ID is UMIN000039949. Figure 1 presents the study protocol for the intervention and control groups.

### Risk evaluation

The interventions in this study were within the regular scope of pharmacy practice, and therefore, we assessed that no additional and fatal risks would arise from participation in the study.

## Results

### Participants

**Fig. 2:**
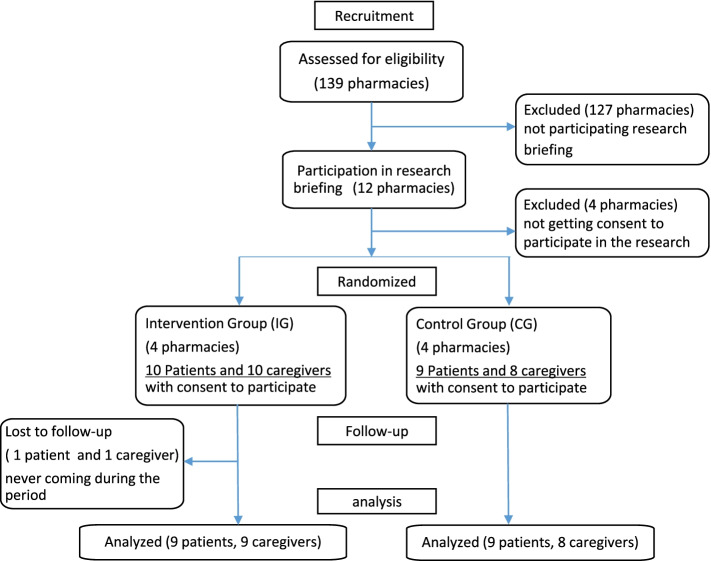
Consort flowchart of participant selection

Of the 139 Nara City pharmaceutical association member pharmacies, 12 attended the study briefings and 8 provided consent to participate in the study. These pharmacies were allocated at a 1:1 ratio to the intervention and control groups. The purpose of the study was explained to the patients with dementia and caregivers visiting the participating pharmacies. Those who gave consent to participate were included in the study. Ten patients and caregivers were randomly assigned to the intervention group, and nine patients and eight caregivers were assigned to the control group (Fig. 2). Nine patients in each group completed the study during the survey period. In each group, two patients were male and seven were female. The primary caregivers included four sons, three daughters, one grandchild, and one spouse in the control group and four sons, three daughters, one grandchild, and one patient in the intervention group (Table 1).

**Table 1:** Baseline demographic and clinical characteristics for each group

Item	IG (*n* = 9)	CG (*n* = 9)
Sex (*n*)		
Male	2	2
Female	7	7
Age (years)		
Mean (SD)	86.1 (5.4)	84.0 (6.6)
Primary caregivers (*n*)		
Patient	0	1
Spouse	1	0
Son	4	4
Daughter	3	3
Grandchild	1	1
Housing circumstances (*n*)		
Single residence	3	4
Living with spouse	1	1
Living with a person other than the spouse	5	4
Number of prescribed medicines (items)		
Mean (SD)	6.9 (4.0)	6.9 (2.9)

*IG* intervention group, *CG* control group, *SD* standard deviation

### Patient-centred outcomes

J-ZBI scores were obtained for the seven caregivers in the intervention group and five in the control group. The mean difference of J-ZBI score in the intervention group was −1.0, and that in the control group was 3.0 (Difference −4.0, 95% CI: −8.563 to 0.563, effect size = 0.68) (Table 2). In the intervention group, J-ZBI scores improved in three subjects, remained unchanged in three, and worsened in one. Meanwhile, in the control group, the J-ZBI score remained unchanged in two subjects and worsened in three. In the intervention group, the caregiver whose J-ZBI score worsened had a patient who was transitioning to tube feeding therapy during the study period. Of the 22 items evaluated in the J-ZBI, 7 scores decreased, and 8 scores increased, resulting in an overall worsening of 1 point.

**Table 2:** Comparison of outcomes in the two groups

	Baseline		After 5 months		Difference		95% CI	Effect size
	IG	CG	IG	CG	IG	CG		
	Mean (SD)	Mean (SD)	Mean (SD)	Mean (SD)	Mean (SD)	Mean (SD)		
J-ZBI score	24.57 (14.85)	15.60 (13.83)	23.57 (15.11)	18.60 (13.22)	−1.00^a^ (2.00)	3.00^a^ (3.74)	−8.563, 0.563	0.68
Caregiver’s understanding of dementia	4.11 (1.05)	3.75 (1.39)	4.44 (1.01)	2.62 (2.13)	0.33^b^ (0.71)	−1.13^b^ (1.89)	−0.148, 3.065	0.57

*IG* intervention group, *CG* control group, *SD* standard deviation, *CI* confidence interval, *J-ZBI* the Japanese version of Zarit Caregiver Burden Interview

^a^Mean differences (after 5 months − baseline) were calculated for the intervention (*n*=7) and the control groups (*n*=5), excluding those items that were not completed

^b^Mean differences (after 5 months − baseline) were calculated for the intervention (*n*=9) and the control group (*n*=8), excluding those items that were not completed

Caregivers’ dementia comprehension scores were obtained for nine participants in the intervention group and eight participants in the control group. The mean difference of comprehension score in the intervention group was 0.33 and that in the control group was −1.13 (Difference 1.46, 95% CI: −0.148 to 3.065, effect size = 0.57) (Table 2). In the intervention group, the scores improved in two subjects, remained unchanged in seven subjects, and did not worsen in any subjects. In the control group, the scores did not improve in any subject, remained unchanged in six subjects, and worsened in two subjects.

The number of problems identified was eighteen for the first time and fifteen for the second time in the intervention group, versus fifteen for the first time and fifteen for the second time in the control group. Prescription changes occurred for eight patients in the intervention group, versus four in the control group (Table 3). Four out of eight prescription changes in the intervention group and one out of four prescription changes in the control group were related to dementia treatment.

**Table 3:** Prescription changes and actions taken

	IG	CG
Change in prescription		
Yes	8	4
No	1	5
Action by family physician and/or care manager		
Yes	3	1
No	6	8

*IG* intervention group, *CG* control group

In the intervention group, six out of nine family physicians and four out of nine care managers responded to The form for request of information issued by the pharmacist. In three cases, pharmacists provided repeat feedback on the family physicians’ responses. For actions, there were eight cases in the intervention group (care plan change: 2, telephone contact from family physician: 2, request for home visiting from family physician: 1, request from care manager to attend care conference: 1, telephone contact from care manager: 2) versus one case in the control group (telephone contact from the care manager (Table 3).We assessed relevant information about three caregivers (cases 1–3) from the intervention group whose J-ZBI scores improved during the study (Table 4) to discuss factors contributing to the reduction of care burden.

**Table 4:** Summary of three cases in which the caregiving burden reduced

	Case 1 (ID 429)	Case 2 (ID 4540)	Case 3 (ID 73570)
**Primary caregiver**	Son	Grandchild	Spouse
**Change in J-ZBI scores**	51 to 50	15 to 10	31 to 29
**Items that improved the J-ZBI score**^**a**^	• Afraid of what the future holds for your relative	• Feel that because of the time you spend with your relative that you do not have enough time for yourself	• Feel that you will be unable to take care of your relative much longer
		• Feel stressed between caring for your relative and trying to meet other responsibilities for your family or work	• Feel you have lost control of your life since your relative’s illness
		• Afraid of what the future holds for your relative	
		• Feel strained when you are around your relative	
		• Feel you have lost control of your life since your relative’s illness	
**Change in comprehension scores**	5 to 5	3 to 5	4 to 5
**Items that improved in comprehension score**^**a**^		• Understand that there are several types of dementia	• Understand that there are several types of dementia
		• Understand that dementia has peripheral symptoms	
**Problems identified by the pharmacist from the Grasp problem sheet**^**a**^	• Take medication at one’s own discretion or not at all	• Not coping with continued medications	• Dissatisfied with the current dosing regimen for the rest of their life
	• Discontinue the medication at your own discretion when your condition changes		• Lack of care for any changes in the patient’s condition while taking the medicines
	• Not being able to ask questions about medications to healthcare providers without hesitation		• Not finding and using the information needed for the patient’s medication
	• Not finding and using the information needed for the patient’s medication		Not asking when you do not know about the medication the patient is using
	• Not coping with continued medications		
	• Not knowing about the medication used by patient and why they are needed.		
	• Not asking when you do not know about the medication the patient is using		
**Reply to the form for request of information**	Reply from family physician and care manager	Reply from family physician and care manager	Reply from family physician and care manager
**Actions by the family physician or care manager**	• The family physician instructed the pharmacist to visit the patient residence.	• Care manager asked pharmacists to participate in care conferences.	• Family physician contacted separately
		• Care manager directly contacted. There was an increase in direct contact from care managers to pharmacists (3 times during the study period).	
**Details of the prescription change**^**a**^	Addition of antitussives	• Donepezil dose increased from 3 to 5 mg.	• Linaclotide and mirabegron tablets were discontinued.
			• Changed from internal vicosulfate solution to tablet

*J-ZBI* the Japanese version of Zarit Caregiver Burden Interview

^a^The items cite the expressions used in the questionnaires

The study identified the following key factors for feasibility:

- Obtaining consent from caregivers is certainly difficult, but possible.
- Pharmacists faced no major problems in completing the survey form while practising pharmaceutical care.
- Many J-ZBI items are time-consuming for caregivers.
- The 5-month follow-up period was too short to obtain clear outcomes or to confirm that the problem had been resolved.

There were no adverse events associated with the interventions in this study.

## Discussion

Although this study did not show statistically significant differences owing to the small sample size, the effect size suggests that active interventions by the community pharmacist may reduce the burden of care for caregivers of people with dementia. Regarding the effect size, the values of 0.1, 0.3, and 0.5 indicated small, medium, and large effects, respectively [34].

In case 1, the pharmacist identified problems related to the ‘awareness of medication’, ‘collaboration with healthcare providers regarding medication’, and ‘motivation to access and utilize information regarding medication’, including discontinuing medication at own direction, not being able to ask questions about medications to healthcare providers without hesitation, and not knowing about the medication used by the patient and why it is required. This information was shared with the family physician and care manager via ‘The form for request of information’, and the pharmacist was subsequently instructed to visit the patient's residence. As a result, it became possible for the pharmacist to provide the necessary information. For instance, they were able to discuss the need and policy for the patient’s specific pharmacotherapy as well as the features of the additional medication; in response, the pharmacist was provided the opportunity to respond to the caregiver’s questions. These interactions may have served to reduce caregiver anxiety as it would be made clear that the necessary support would be available when required, while also providing the pharmacist with a better understanding of patient and caregiver living circumstances, allowing them to tailor the patient’s care accordingly.

In case 2, the pharmacist identified problems related to ‘motivation to access and utilize information regarding medication’, particularly related to continuation of medication. Subsequently, through information sharing via ‘The form for request of information’, the frequency of contact between the care manager and pharmacist increased, leading to pharmacist participation in the care conference of the patient in question. Care conferences are organized by care managers and include the gathering of healthcare providers and caregivers to identify specific issues and requests related to the patient’s medical care, while assessing the support measures. During the care conference, the pharmacist involved in the patient’s care is afforded the opportunity to respond directly to the caregiver’s questions and concerns, thus improving the caregiver’s understanding of dementia. The conference also allows caregivers to experience the benefits of a multi-profession support system in the care of the patient, as well as the support system’s capacity to potentially reduce stress and anxiety associated with care. Additionally, in this specific case, treatment with donepezil was effectively continued after a prescription change. This discussion is also supported by previous reports claiming that the addition of pharmacists to care teams for people with dementia leads to a reduction in the care burden for caregivers [35].

In case 3, issues related to ‘awareness of medication’ and ‘motivation to access and utilize information regarding medication’ were identified, including dissatisfaction with the current dose for the rest of the patient’s life, lack of regard for any changes in the patient’s condition while taking the medicine, not finding and using the information needed for medication, and not asking questions when not knowing what the patient is using. Subsequently, information obtained through the ‘The form for request of information’ increased the frequency of contact between the family physician and the pharmacist and revealed that the patient was reluctant to use the patch medication, while the caregiver believed that there were no side effects to it. No changes were made to this prescription during the survey period; however, two other medications were discontinued, and the route for administering constipation medication was changed for ease of management. Enhanced collaboration between the physician and pharmacist may have improved the caregivers’ comprehension of dementia and reduced the caregivers’ sense of constraint due to the care provided. These findings were consistent with previous reports that have indicated that effective physician-pharmacist communication contributes to a better understanding of patient needs [36], while information sharing among families, physicians, care managers, and other professionals involved in care contributes to a reduction in the care burden [37].

In the three case studies discussed, we believe that the active information gathering from other professionals conducted by the pharmacists in the intervention group enabled the identification and individualization of problems contributing to the burden of care and improved the caregivers’ comprehension of medication. This conclusion is supported by the caregivers’ improved comprehension of the dementia medication, allowing for better medication control and improved medication adherence [38], and by the reported increase in the caregivers’ self-efficacy and reduction in the burden of caregiving [39–41]. Meanwhile, it has also been reported that psychological interventions for caregivers by healthcare professionals contribute to reduced care burden [42, 43]. Therefore, in the future, it is important for pharmacists to actively conduct information gathering for people with dementia, cooperate with other professionals, share information, and conduct assessments. It will also be important for pharmacists to gain familiarity with programmes to support self-care for caregivers, such as cognitive behavioural therapy, and to seek and practice communication and engagement with caregivers.

However, the small sample size and short follow-up period represent limitations of this study. Additionally, counselling conducted with pharmacists after study completion revealed that while the presented protocol was believed to be feasible, the explanatory document provided to the pharmacists requires improvement. Specifically, a more thorough explanation regarding the study design and commitments is required for the pharmacies to provide consent for participation. It was also noted that the intervention process requires simplification. Accordingly, the proposed changes include simplifying the methods by which data is collected, using a shorter version of the J-ZBI (J-ZBI 8) [44], and prolonging the follow-up period (e.g. matching the care manager to the 6-month interval between periodic reviews of the care plan).

### Generalizability (applicability) of pilot trial methods and findings to future definitive trial and other studies

The feasibility of this trial was verified. However, large-scale studies are required for further standardization and simplification of the protocol to generalize these results. Based on the findings from this study, the following points should be considered in the main study.

- The number of participants obtained per pharmacy was low, so the number of pharmacies needs to be increased.
- Improvements were needed in the various survey forms and in the explanatory documents for caregivers.
- The intervention process needs to be simplified in order to conduct a large study.
- Since the 22-item version of the J-ZBI is burdensome to fill out, it is recommended that an 8-item version be used in the main study.
- The follow-up period should be extended to a minimum of 6 months.

Thus, although this trial focused on dementia, we believe that the protocol can be restructured to be applied to other diseases.

## Conclusion

The protocol presented was considered feasible, but the intervention process needs to be simplified in order to conduct a large study. Also, improvements are needed in the various survey forms and in the explanatory documents for caregivers. This was a preliminary study, and due to the low sample size, we were not able to show statistically significant differences in J-ZBI scores between the two groups, although we were able to obtain sufficient effect size.

Active intervention by community pharmacists for patients with dementia is important not only for the appropriateness of medication therapy, but also for its potential contribution to improving the quality of life of patients and caregivers by reducing the burden of care. In the future, we plan to conduct a study with a larger number of participants to confirm the results of the current study.

## Supplementary Information

**Additional file 1: Appendix 1.** Basic Information Sheet. **Appendix 2.** Understanding of dementia survey sheet. **Appendix 3.** Grasp problems sheet. **Appendix 4.** The form for request of information (Family Physician). **Appendix 5.** The form for request of information (Care Manager).

## Data Availability

The datasets used and/or analysed during the current study are available from the corresponding author on reasonable request.

## Abbreviations

IG: Intervention group
CG: Control group
SD: Standard deviation
J-ZBI: Japanese version of the Zarit Caregiver Burden Interview
FP: Family physician
