# Supplementary material for: Impact of interventions by a community pharmacist on care burden for people with dementia: development and randomized feasibility trial of an intervention protocol
Source: Pilot Feasibility Stud. 2022 Jun 2;8:118. doi: 10.1186/s40814-022-01071-7 (PMC9161485; doi:10.1186/s40814-022-01071-7)
Supplement: Supplementary file 1 — Additional file 1: Appendix 1. Basic Information Sheet. Appendix 2. Understanding of dementia survey sheet. Appendix 3. Grasp problems sheet. Appendix 4. The form for request of information (Family Physician). Appendix 5. The form for request of information (Care Manager). [file 40814_2022_1071_MOESM1_ESM.docx]

**Appendix 1**

**A: Basic Information Sheet Date 　　　　　(the first and __time)**

To be filled during the first visit and every subsequent visit (complete the form during every visit if the interval between visits is more than one month, or every month if the interval is less than one month).

| Pharmacy name | | | | | Patient ID | | **①**1. Male, 2. Female  ②years old | | |
| --- | --- | --- | --- | --- | --- | --- | --- | --- | --- |
| **③ Primary caregiver** | 1. Individual 2. Spouse 3. Son 4. Daughter 5. Marriage 6. Son-in-law 7. Other family 8. Counselor  9. Care Manager 10. Visiting Caregiver 11. Visiting Nurse 12. Other Participants ( ) | | | | | | | | |
| **④ Residential environment**  1. Living alone 2. Living with a spouse 3. Living with a family member other than the spouse 4. Care homes for the elderly with nursing care services 5. Group home 6. Other facilities | | | | | | | | | |
| **⑤ Content of prescriptions by medical institutions providing treatment for dementia**  (Dispensing record may be attached with the personal information deleted.) | | | | | | **⑥ Prescriptions from medical institutions other medical institutions**  Clinical department: | | | |
| 1. Drug name (specification) | | 2. Daily dose | 3. Usage | | 4. Number of days | a. Drug name (specification) | b. Daily dose | c. Usage | d. Number of days |
|  | |  |  | |  |  |  |  |  |
| **Please answer the following after the second visit.** | | | | | | | | | |
| **⑦ Whether or not the prescription has been modified since the previous visit** | | | | 1. Yes 　2. No | | | | | |
| ⑦-1 If yes, please choose the appropriate option(s) from the right.  (Multiple answers allowed) | | | | a. Prescription drugs decreased b. Prescription drugs increased c. Prescription days changed  d. e. Others ( ) for which prescription medication has been changed | | | | | |
| **⑧Was any action taken by the family physician and/or care manager?** | | | | 1. Yes 　2. None | | | | | |
| ⑧-1　If yes, please choose the appropriate option(s) from the right.  (Multiple answers allowed) | | | | a. My family physician requested me to check the remaining drugs.  b. My family physician asked the pharmacist to provide information.  c. My family physician instructed the pharmacist to visit patient’s home.  d. The care manager asked me to participate in the care conference.  e. The care manager visited my pharmacy.  f. Other ( ) | | | | | |

**Appendix 2**

**B: Understanding of dementia survey sheet**

**The pharmacist should interview and fill out this survey with the person who came to pick up the medicines.**

**Date of completion of patient ID (at the end of the first survey)**

**Primary caregiver: ( )**

**Please check the primary caregiver's understanding of dementia.**

| 1. Understand that the progression of dementia can be slowed by treatment and care | **1. Yes 0. No** | If any question is answered as “no,” it is assumed that there is a problem in the understanding of dementia |
| --- | --- | --- |
| 2. Understand that the progression of symptoms of dementia can be slowed down by taking medications | **1. Yes 0. No** |  |
| 3. Understand that dementia has ‘peripheral symptoms’ in which behavioral and mental disorders occur | **1. Yes 0. No** |  |
| 4. Understand that there are several types of dementia, including Alzheimer's type and multi-infarct type | **1. Yes 0. No** |  |
| 5. Understand that measures against lifestyle related diseases are effective in preventing dementia. | **1. Yes 0. No** |  |

**Appendix 3**

**C: Grasp problems sheet**

**To be filled out by the pharmacist after interviewing the caregiver**

**Date of completion of patient ID (at the end of the first survey)**

**Primary caregiver:(　　　　　　　)**

**Please mark the primary caregiver on the following criteria.**

1. Regarding the primary caregiver's “awareness of medication.” Please circle a number 1−5 that most closely reflects the caregiver’s understanding of dementia based on the answers given.

| a. Do you remember how to take/give the medicine (when and how much)? | 5. Remember all 4. Remember most 3. Remember about half.  2. Don't remember well 1. Do not remember at all. | If you have answered 1 or 2 for any of a~f questions, conclude that there is a problem with awareness of medication. |
| --- | --- | --- |
| b. Are you taking the medicine as directed?  (Or are you able to give the patient the medicine as directed?) | 5. Take/give all medication as per the recommended dose and regimen  4. Sometimes forget to take it as directed, but I notice it.  3. Unable to take/give the medicines as directed several times a week.  2. Frequently cannot take/give the medicine as directed  1. Take/give medication at one’s own discretion or not at all |  |
| c. Do you know what the medicines prescribed do (what symptoms do they alleviate)? | 5. Know all 4. Know most 3. Know about half.  2. Don't know well 1. Do not know anything. |  |
| d. How would you respond if there were a change in the patient’s physical condition while taking the medicines? | 5. Condition known beforehand and respond by yourself  4. Notify healthcare providers when you are aware  3. Don't inform healthcare providers even if you are aware  2. Discontinue the medication at your own discretion.  1. Don't care about it. |  |
| e. Are you taking/giving the medicine and do you know why? | 5. Know all 4. Know most 3. Know about half.  2. Don't know well 1. Do not know anything. |  |
| f. How would you feel if the current dosing regimen were to continue for the rest of the patient’s life? | 5. Satisfied 4. Mostly satisfied 3. Neither  2. Dissatisfied 1. Very dissatisfied |  |

2. Please tell us about the level of collaboration or any aggressiveness of the primary caregiver regarding medication, and their degree of satisfaction and harmonization with the medication.

| **I. Collaboration with healthcare providers regarding medication** | **4. Strongly agree. 3. Agree. 2. Disagree. 1. Strongly disagree.** | If you have answered 1 or 2 to any of the items, conclude that  There is a problem in coordination, aggressiveness, and harmony attitude, and harmony in medication. |
| --- | --- | --- |
| a. Share your own thoughts and goals with doctors and other healthcare professionals.  b. Share medication-related information and the patient’s progress over the course of treatment with medical professionals, such as physicians.  c. Ask questions about medications to healthcare providers without hesitation. | **4 ・ 3 ・ 2 ・ 1**  **4 ・ 3 ・ 2 ・ 1**  **4 ・ 3 ・ 2 ・ 1** |  |
| **II. Motivation to access and utilize information regarding medication** | **4. Strongly agree. 3. Agree. 2. Disagree. 1. Strongly disagree.** |  |
| 1. Finding and using the information needed for the patient’s medication. 2. Cope to continue medications (e.g., contrivance in everyday life). 3. Report adverse reactions/allergic symptoms to the drug and any unusual symptoms. 4. Know about the medications used by patients and why they are needed. 5. Ask when you do not know about medications the patient is using. | **4 ・ 3 ・ 2 ・ 1**  **4 ・ 3 ・ 2 ・ 1**  **4 ・ 3 ・ 2 ・ 1**  **4 ・ 3 ・ 2 ・ 1**  **4 ・ 3 ・ 2 ・ 1** |  |
| **III. Agreement with taking medications and their fit with patient’s lifestyle** | **4. Strongly agree. 3. Agree. 2. Disagree. 1. Strongly disagree.** |  |
| 1. I am satisfied with the patient's need for medicine. 2. The patient's use of medicine is part of his or her lifestyle, such as eating or brushing his or her teeth. 3. There is no resistance to getting help from family members or people around me, such as to speak about medications. | **4 ・ 3 ・ 2 ・ 1**  **4 ・ 3 ・ 2 ・ 1**  **4 ・ 3 ・ 2 ・ 1** |  |

**Appendix 4**

The form for request of information (Family Physician)

Name of medical institution 　　　　　　　　　　　　　 Date

To Family Physician

Pharmacy name　　　　　　　Pharmacist name

Address

　　　　　　　　　　　　　　　Telephone/Fax

We gave medication guidance to the following patients at our regulatory pharmacy. I believe that we would be able to improve patient compliance and respond to patient concerns more effectively if you could share the treatment plans, prescription policy, and opinions of the family physician.

| Patient name Male/Female  Date of birth |
| --- |

| Primary disease |
| --- |
| Please let me know if you have any information such as clinical test data for the following items.  □ Items for clinical data we required  Please let me know if there are any data on the patient that you used to diagnose this patient, such as clinical test data other than that mentioned above. |
| Do you cooperate to other occupations (such as care managers and nursing care providers)?  1. □ Provide and share information with each other and cooperate  2. □ We provide information, but do not receive information from the other professionals.  3. □ It is difficult to cooperate.  4. □ There is no cooperation.  <Please let us know if you have a problem with cooperation with other occupations with regards to this patient.> |
| Please tell us the details of guidance offered to family members and primary caregivers, as well as the future treatment plans. |
| Please let me know if you have any requests for the pharmacy (e.g. request pharmacist to visit the patient’s home to check the remaining drugs, to give instructions to the family, to do pharmacist home-visiting service, etc.) |

**Appendix 5**

The form for request of information (Care Manager)

Care management office Date

Responsible care manager

Regional Comprehensive Support Center

Pharmacy name Pharmacist name

Address

　　　　　　　　　　　　　　　Telephone/Fax

The following patients have been offered medication guidance at our regulatory pharmacy. I believe that better patient compliance and response to patient concerns would be possible if the person in charge shared the following information.

| Patient name: Male/Female  Date of birth |
| --- |

| Please tell me the main disease that resulted in the certification of nursing care. |
| --- |
| Please tell me the degree of care required, the results of the care assessment, and the policy regarding the care plan. |
| Are you in contact with the family physician?  1. □ Provide and share information with each other to cooperate.  2. □ Provide information, but not receive information from the family physician.  3. □ It is difficult to collaborate.  4. □ There is no collaboration.  <Please let me know if there are any problems with collaboration and cooperation with the doctor regarding this patient> |
| Challenges and future policies in creating care plans |
| Please let me know if you have any requests for the pharmacy. |
